# Supplementary material for: Selective targeting of non-centrosomal AURKA functions through use of a targeted protein degradation tool
Source: Commun Biol. 2021 May 28;4:640. doi: 10.1038/s42003-021-02158-2 (PMC8163823; doi:10.1038/s42003-021-02158-2)
Supplement: Supplementary file 2 — Description of Additional Supplementary Files [file 42003_2021_2158_MOESM2_ESM.pdf]

Description of Additional Supplementary Files

File name: Supplementary Data 1

Description: Image quantification data used to plot graphs shown in Figures 1-7.
