# Supplementary material for: Selective targeting of non-centrosomal AURKA functions through use of a targeted protein degradation tool
Source: Commun Biol. 2021 May 28;4:640. doi: 10.1038/s42003-021-02158-2 (PMC8163823; doi:10.1038/s42003-021-02158-2)
Supplement: Supplementary file 4 — Reporting Summary [file 42003_2021_2158_MOESM4_ESM.pdf]

nature research

Corresponding author(s): Catherine Lindon

Last updated by author(s): 15/4/21

Reporting Summary

Nature Research wishes to improve the reproducibility of the work that we publish. This form provides structure for consistency and transparency in reporting. For further information on Nature Research policies, see our Editorial Policies and the Editorial Policy Checklist.

Statistics

For all statistical analyses, confirm that the following items are present in the figure legend, table legend, main text, or Methods section.

n/a	Confirmed	
☐	☒	The exact sample size (n) for each experimental group/condition, given as a discrete number and unit of measurement
☐	☒	A statement on whether measurements were taken from distinct samples or whether the same sample was measured repeatedly
☐	☒	The statistical test(s) used AND whether they are one- or two-sided<br>*Only common tests should be described solely by name; describe more complex techniques in the Methods section.*
☒	☐	A description of all covariates tested
☐	☒	A description of any assumptions or corrections, such as tests of normality and adjustment for multiple comparisons
☐	☒	A full description of the statistical parameters including central tendency (e.g. means) or other basic estimates (e.g. regression coefficient) AND variation (e.g. standard deviation) or associated estimates of uncertainty (e.g. confidence intervals)
☒	☐	For null hypothesis testing, the test statistic (e.g. F, t, r) with confidence intervals, effect sizes, degrees of freedom and P value noted<br>*Give P values as exact values whenever suitable.*
☒	☐	For Bayesian analysis, information on the choice of priors and Markov chain Monte Carlo settings
☒	☐	For hierarchical and complex designs, identification of the appropriate level for tests and full reporting of outcomes
☒	☐	Estimates of effect sizes (e.g. Cohen's d, Pearson's r), indicating how they were calculated

Our web collection on statistics for biologists contains articles on many of the points above.

Software and code

Policy information about availability of computer code

Data collection	No custom code was used for data collection.
Data analysis	No custom code was used for data analysis.

For manuscripts utilizing custom algorithms or software that are central to the research but not yet described in published literature, software must be made available to editors and reviewers. We strongly encourage code deposition in a community repository (e.g. GitHub). See the Nature Research guidelines for submitting code & software for further information.

Data

Policy information about availability of data

All manuscripts must include a data availability statement. This statement should provide the following information, where applicable:

- Accession codes, unique identifiers, or web links for publicly available datasets
- A list of figures that have associated raw data
- A description of any restrictions on data availability

Data availability statement is included in manuscript

Field-specific reporting

Life sciences study design

All studies must disclose on these points even when the disclosure is negative.

Sample size	The number of cells measured for each live cell experiment was determined by how many positions the automated stage could visit at each time point. For some experiments where sample size was low, data from separate repeats of the experiment were pooled, as indicated in figure legends.
Data exclusions	In datasets containing outliers (because of very high protein expression in transient transfection experiments, or very low expression giving rise to low signal:noise), expression limits were imposed on data analysed within the experiment, excluding some datapoints.
Replication	For immunofluorescence analyses, two or three cover-slips were used for each condition. Images were taken of all cover-slips and compared to check that staining intensities were similar. Only comparable cover-slips were analysed, and data pooled for each condition. Number of experimental repeats (two, three or four) for all experiments are indicated in figure legends.
Randomization	N/A
Blinding	Scoring of phenotypes by immunofluorescence was carried out blind.

Reporting for specific materials, systems and methods

We require information from authors about some types of materials, experimental systems and methods used in many studies. Here, indicate whether each material, system or method listed is relevant to your study. If you are not sure if a list item applies to your research, read the appropriate section before selecting a response.

Materials & experimental systems

n/a	Involved in the study	
☐	☒	Antibodies
☐	☒	Eukaryotic cell lines
☒	☐	Palaeontology and archaeology
☒	☐	Animals and other organisms
☒	☐	Human research participants
☒	☐	Clinical data
☒	☐	Dual use research of concern

Methods

n/a	Involved in the study	
☒	☐	ChIP-seq
☒	☐	Flow cytometry
☒	☐	MRI-based neuroimaging

Antibodies

Antibodies used	AURKA mouse mAb (1:1000; Clone 4/IAK1, BD Transduction Laboratories), phospho-Aurora A (Thr288)/Aurora B (Thr232)/Aurora C (1:1000; clone D13A11 XP® Rabbit mAb, Cell Signalling), rabbit polyclonal TPX2 antibody (1:1000; Novus Biological), AURKB rabbit polyclonal antibody (1:1000; Abcam ab2254), mouse mAb Cyclin B1 (1:1000; BD 554177), rabbit polyclonal beta-tubulin (1:2000; Abcam ab6046), GAPDH rabbit mAb (1:400; Cell Signaling Technology #2118), TACC3 rabbit polyclonal antibody (1:1000; gift from F. Gergely), CEP192 affinity-purified rabbit polyclonal antibody (1:1000; Gift from L. Pelletier)
Validation	Antibody validation information is on manufacturer's website. TACC3 and CEP192 antibodies were confirmed by localization in IF and CEP192 siRNA.

Eukaryotic cell lines

Policy information about cell lines

Cell line source(s)	Parental hTERT-RPE1-FRT/TO cells and hTERT-RPE1-cyclin B1-VenusKI were gift from Jonathon Pines Lab, Gurdon Institute, Cambridge. All AURKA and AURKB lines were derived in our lab, except for hTERT-RPE1-AURKA-VenusKI cells, which were a gift from Jorg Mansfeld lab, TU Dresden. U2OS FZR1 KO cells were made in our lab.
Authentication	All lines authenticated prior to this study.
Mycoplasma contamination	All cells tested negative for mycoplasma contamination
Commonly misidentified lines (See ICLAC register)	N/A
